# Supplementary material for: Learning curve and surgical outcome of robotic assisted colorectal surgery with ERAS program
Source: Sci Rep. 2022 Nov 29;12:20566. doi: 10.1038/s41598-022-24665-w (PMC9709162; doi:10.1038/s41598-022-24665-w)
Supplement: Supplementary file 1 — Supplementary Information 1. [file 41598_2022_24665_MOESM1_ESM.zip › LSC Raw data-Ñ[▒K/064/N_ñw╝╨░O▒Kñσ.pdf]

W86 / W86

臺中榮民總醫院
手術室護理記錄

[redacted]

基本資料

- 室別： 開刀房-06室，手術科別： CRS，手術類別： 常規，日期： 2020/02/06
- 進入時間： 1435 ； 麻醉開始時間： 1440 ； Time Out時間： 1545 ； 劃刀時間： 1546 ； 手術結束時間： 1910 送出時間： 1915 ；
- 麻醉方式： GE
  診斷：Malignant tumor of rectum
  手術名稱：Low anterior resection with colo-anal anastomosis
  Laparoscopy
  手術局部用藥：nil
  術前預防性抗生素：
  - 抗生素名稱一： Veterin 1GM IVD由麻醉護理人員執行
  - 時間： 1520
  - 上次簽章: 劉裴瑀

  ，追加預防性抗生素：
- 手術醫師： 蔣鋒帆，許家榮，黃俋霖，楊涵中，陳思如
- 麻醉醫師： VS 張詒婷，麻醉醫師： VS 劉恆豪
-
  - 刷手護士A ： 呂昭蓉，時間： 1440，接替刷手一： 周桂英，時間： 1500，接替刷手二： 劉裴瑀，時間： 1800
  - 流動護士A ： 劉裴瑀，時間： 1440，接替流動一： 陳有容，時間： 1730

皮膚狀況

A-V Shunt： 無
術前： 完整
記錄者： 上次簽章: 劉裴瑀
術後： 完整
記錄者： 上次簽章: 陳有容

手術準備

手術臥式 單一手術臥式

-
  - 手術臥式： 截刀臥式
  - 使用附件： 溫毯 38 ℃，腳架
  - 皮膚準備： 10%Aq Betadine，Hibiscrub，其它： 2%chlorhexidine
  - 電燒： 使用電燒: 電極片位置： 右大腿，記錄者： 上次簽章: 劉裴瑀

止血帶T： 無

植入物

無

標本送檢

- 細菌培養 無
- 細胞學 無
- 冰凍切片 無
- 病理標本
  組織名稱一：Rectum
  ,
  組織名稱二：Distal cut end
  ,
  組織名稱三：IMA root
- 截肢 無

標本交付 標本： 3 袋，標本： 0 盒，標本： 0 瓶，細胞學： 0 管，Culture Tube： 0 管，被交付醫師： R 楊涵

中，

- 記錄者： 上次簽章: 陳有容
- 時間： 1910

傷口術後數料

傷口數料：

- 請於圖上標示部位：

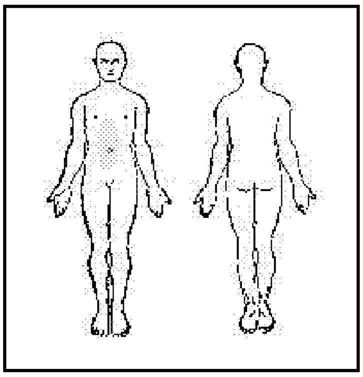

- 傷口數料： mepore

導管

導管	天數	導管資訊	插入日期	插入時間	更換日期	拔除日期	拔除時間	拔除原因	預計拔除/更換	預計拔除/更換日期
A-line (1)	1	號碼:20 G; 部位:左饒動脈;	2020/02/06	1450						
Sump NG Tube(1)	1	號碼:16 Fr.; 深度:50cm; 部位:胃;	2020/02/06	1515						
Double Lumen CVC(1)	1	號碼:8 Fr.; 深度:15cm; 部位:subclavian (左); 象限:LU;	2020/02/06	1510						
Foley(1)	1	號碼:16 Fr.; 部位:膀胱; 註記:Balloon 10ml;	2020/02/06	1455						

用物計數

- 手術中出血量： 少 c.c.
- 手術中尿量： 250 c.c.
-
    - 其它： 無

用物計數： 多切口同時縫合

- 1.器械： 正確，2.縫針： 正確，3.刀片： 正確，4.紗布： 正確，5.腹部墊： 未使用，6.紗球： 未使用，7.PEANUTS ： 未使用，8.紗條： 未使用，9.絲帶： 未使用，10.針頭： 未使用，11.Vessel loop： 未使用，12.Boots： 未使用，13.Jelco： 未使用，14.棉條： 未使用，
    - 第一次：
        - 刷手護士 ： 劉裴瑀
        - 流動護士： 上次簽章: 陳有容
        - 時間： 1830

    ，
    - 第二次：
        - 刷手護士 ： 劉裴瑀
        - 流動護士： 上次簽章: 陳有容
        - 時間： 1845

    ，
    - 第三次：
        - 刷手護士 ： 劉裴瑀
        - 流動護士： 上次簽章: 陳有容
        - 時間： 1850

備註

備註： 腹腔鏡NO.1

- 記錄者： 上次簽章: 劉裴瑀
- 時間： 1625

填寫護理人員 陳有容

正式摘要

	W86 / W86

doctorRank:B
